# Antiobesity Effect of *Codonopsis lanceolata* in High-Calorie/High-Fat-Diet-Induced Obese Rats

**DOI:** 10.1155/2013/210297

**Published:** 2013-05-30

**Authors:** Hye-Kyung Choi, Eun-Kyung Won, Young Pyo Jang, Se-Young Choung

**Affiliations:** ^1^Department of Preventive Pharmacy and Toxicology, College of Pharmacy, Kyung Hee University, Seoul 130-701, Republic of Korea; ^2^Division of Pharmacognosy, College of Pharmacy, Kyung Hee University, Seoul 130-701, Republic of Korea

## Abstract

The antiobesity effects of *Codonopsis lanceolata* (CL) were evaluated in a high-calorie/high-fat-diet (HFD-) induced obesity rat model and 3T3-L1 cells. The Sprague-Dawley male rats were fed a normal diet (ND) or a HFD for a period of 12 weeks. The rats were subdivided into groups: ND, ND + wild *Codonopsis lanceolata* (wCL) (900 mg/kg/day, p.o.), ND + cultivated *Codonopsis lanceolata* (cCL) (900 mg/kg/day, p.o.), HFD, HFD + wCL (100, 300, or 900 mg/kg/day, p.o.), HFD + cCL (100, 300, or 900 mg/kg/day, p.o.), and HFD + sibutramine. The body weight gains of the administered HFD + CL (wCL or CCL) were lower than those of the rats fed with only the HFD group. Moreover, the weight of adipose pads and the serum levels of triglycerides, total cholesterol, and low density lipoprotein cholesterol in the group administered HDL + CL were significantly lower than in the HFD group. The inhibitory effect of lipid accumulation in 3T3-L1 cells was measured by Oil Red O staining and reverse transcription-polymerase chain reaction (RT-PCR). Treatment of 3T3-L1 cells with wCL inhibited lipid accumulation and expression of C/EBP*α* and PPAR*γ*. These results suggest that CL has a great potential as a functional food with anti-obesity effects and as a therapeutic alternative in the treatment of obesity.

## 1. Introduction

As obesity has increased in the population, multiple methods of dieting to achieve weight loss have been introduced. Among these diets, controlling calorie intake and increasing calorie consumption expenditure through a proper exercise have been considered as the ideal. However, a large number of people depend on fasting and the consumption of diet food and medicine to control their weight. This tendency has affected food consumption trends and has led to an increase in the consumption of functional foods for antiobesity, leading to a strong demand for the development of new sources of functional foods and related studies on their efficacy. 

In recent years, there have been many efforts to find promising functional foods that can be used on a daily basis to improve physical constitution. A good example of this is *Clerodendron glandulosum* Coleb. This herb, found in north eastern states of India [1], is a folklore medicine consumed by urban and tribal populace of Manipur against obesity, diabetes, and hypertension [2, 3]. Recently, it has been reported that aqueous extracts from *Clerodendron glandulosum* Coleb. can regulate high-fat-diet induced hyperlipidemia in rats [3], improve fructose induced insulin resistance and hypertension [4], and prevent high-fat-diet-induced hepatic steatosis [5].

As part of these recent endeavors, this study was performed to investigate the influence of the water extracts of wild and cultivated *Codonopsis lanceolata *on the serum and body fat levels of rats fed a high-fat-diet. *Codonopsis lanceolata *(CL) is a perennial plant in the family of Campanulaceae, often called *Baishen* or *Shashen* in China. CL can be classified as *Bei Sha Shen*, which shows a red surface and has a thick root and many rootlets, or *Nam Sha Shen*, which has a light brown surface with a long and fine root. Wild CL (wCL) collected from an alpine district of Gangwondo and cultivated CL (cCL) from Yeongcheon of Gyeongbuk in Korea were used for this study. CL has long been used as traditional fork medicine to treat asthma, phthisis, tuberculosis, bronchitis, dyspepsia and psychoneurosis in Korea, Japan, and China [6–8]. The root of CL, which is composed of various active components including tannins, saponins, polyphenolics, alkaloidss, essential oils, and steroids [6–9], has pharmacological properties including antiobesty, antioxidant, antimicrobial, anti-inflammatory, and immunomodulatory activites [10–13]. Recent studies have documented the biological activities of CL in which water extracts effectively suppressed the high-fat-diet-induced accumulation of neutral fat and total cholesterol in serum and the liver in the experimental model [13]. The ethanol extracts of CL demonstrated very strong antioxidant effects comparable to those of ginseng extract [14]. In addition, Lee et al. (1995) and Lee (2002) reported that the aqueous extracts of CL acted on the immune system in direct and indirect manners and increased the cellular immune reaction [15, 16]. Another report showed that 70% MeOH extracts of CL increased the number of helper T cells [17]. Our study was performed to evaluate the antiobesity activity of CL from different sources: the wild type grown naturally on the mountain and the cultivated type. This study attempted to analyze and compare the potential of wCL and cCL as promising resources for the development of a functional food to control obesity.

## 2. Materials and Methods

### 2.1. Plant Material and Reagents

wCL (voucher specimen number SNBA200505262013, the National Biospecies Knowledge Information System, Korea) produced from the Youngmun Mt. Corporation of the Agricultural Association and cCL (voucher specimen number KBNA200408101015, the National Biospecies Knowledge Information System, Korea) obtained from Yeongcheon of Gyeongbuk were used for this study. These two products were ground separately, and 1 liter of distilled water was added to 100 g of each of the ground wCL and cCL samples, followed by three extraction at 100°C over 4 hours using a shaking extraction method. The extracts were filtered, vacuum-dried, and then freeze-dried. The final yields of the extract from the dried samples of wCL and cCL were 7.2%, respectively.

Test kits for measuring the total cholesterol, triglyceride, and HDL-cholesterol in the serum were purchased from Asan Pharmaceutical, Korea. Glucose and all other reagents were purchased from Sigma, USA. 

### 2.2. Preparation of Samples for LC-MS Analysis

The powder of water extracts from roots of the wCL (200 mg) was dissolved in 100 mL of distilled water, which was then partitioned three times with the same volume of ethyl acetate [18]. The water fraction was evaporated to dryness under a vacuum. The dried samples were dissolved in distilled water (1 mg/mL) and then subjected to a reverse phase HPLC column.

### 2.3. HPLC-ESI-MS Analysis

The HPLC column used was an Atlantis T3 RP18 (4.6 × 150 mm, 3 *μ*m; waters) equipped with an Atlantis T3 RP18 guard column (4.6 × 20 mm, 3.5 *μ*m, waters). The mobile phases were constituted with solvent A (distilled water) and solvent B (acetonitrile). The linear gradient program was as follows: 0–60 min 5%–50% B; 60–90 min 50%–70% B; 90–120 min 70%–100% B, running at a constant flow rate of 0.2 mL/min (P680 Pump, DIONEX). The injection volume of the sample was 10 *μ*L for every injection. A UVD340U diode array detector performing the wavelength scanning from 210 to 365 nm and an ASI-100 automatic injector were used. The operating conditions of the ESI ion source (Ion Sense, Tokyo, Japan) coupled with a JMS-T100TD (AccuTOF-TLC) in the positive ion modes were a desolating chamber at 250°C and an orifice at 180°C. The first orifice lens was set to 85 V, and the ring lens voltage was 10 V. The TOF-MS was set with a peak voltage of 1500 V with a detector voltage of 2200 V. The nebulizing and desolvating gases were set to 1.0 L/min and 1.5 L/min, respectively.

### 2.4. Animals and Treatment

Six-week-old male Sprague-Dawley rats, which is a good animal model for studying the obesity in humans [19, 20], were obtained from Samtako (Osan, Korea) and raised under constant conditions (temperature: 20 ± 2°C; humidity: 40%–60%; lights: 12 hours of light/dark cycle) for 7 days. Body weights ranged between 200 and 210 g. The rats were provided commercially available normal diet (Jongang Lab Animal, Seoul, Korea), prior to the dietary manipulation. All of the samples were administered at a specific time.

During the study period, two diets were used: the normal diet or the high-calorie/high-fat-diet which modified the AIN-76 dietary composition [21], as shown in Table 1. A high-calorie/high-fat-diet, which is commonly used in nutritional experiments as a strategy to induce fat deposition and overweight conditions in SD rat [22, 23], was applied for six weeks to induce obesity. The body weight of these rats increased more than 125% compared to that of the rats with normal diet. The rats of the normal test group were fed with a normal diet (ND) for six weeks, while the rats of the control group were fed with a high-calorie/high-fat-diet (HFD) for six weeks to elicit diet-induced obesity. 

Then the animals were subdivided into 11 groups (*n* = 10)—ND, ND + wCL (900 mg/kg), ND + cCL (900 mg/kg), HFD, HFD + wCL (100, 300, or 900 mg/kg), HFD + cCL (100, 300, or 900 mg/kg) group and HFD + sibutramine—and orally administered with CL or sibutramine once per day for another 6 weeks (Table 2) [23]. All experimental procedures were carried out in accordance with the protocol that was approved by the Institutional Animal Care and Use Committee guideline of Kyung Hee University.

### 2.5. Specimen Collection

Blood was collected from the abdominal aorta of the rat anesthetized using CO_2_ gas after fasting for 12 hours on the last day of the 6-week feeding period. The collected blood was processed using a microcentrifuge method, and the serum was stored in a freezer at −70°C. The retroperitoneal, epididymal, and brown fat pads were removed, rinsed with phosphate-buffered saline, and then weighed.

### 2.6. Blood Sampling and Plasma Assay

The total cholesterol (TC), triglyceride (TG), and high density lipoprotein (HDL)-cholesterol of the serum were measured using a reagent kit (Asan Pharm., Hwaseong, Korea) based on the enzymatic colorimetric method. The level of low density lipoprotein-(LDL-) cholesterol was determined using the Friedewald formula, where LDL-cholesterol = TC − HDL-cholesterol − (TG/5).

### 2.7. Cell Culture and Differentiation

3T3-L1 cells were cultured to confluence in 6 plates in Dulbecco's Modified Eagle's Medium (DMEM) supplemented with 10% (v/v) Bovine Calf Serum (BCS). On the second day of postconfluence (designated as day 0), the cells were induced to differentiate DMEM supplemented with 10% (v/v) FBS (Fetal Bovine Serum), 1 *μ*M dexamethasone, 0.5 mM isobutylmethylxanthine and 5 *μ*g/mL insulin. After 48 hours, the media were replaced with DMEM supplemented with 10% FBS and 1 *μ*g/mL insulin. The cells were subsequently refed every 48 hours with DMEM supplemented with 10% FBS.

### 2.8. Oil Red O Staining

3T3-L1 cells were washed twice with ice-cold phosphate-buffered saline (PBS), fixed with 10% formalin and then stained with 0.5% Oil Red O.

### 2.9. Reverse Transcriptase-PCR Analysis of C/EBP*α* and PPAR*γ*

Total RNA was isolated from cells using the Trizol reagent. One microgram of total RNA was used for single strand cDNA synthesis. Reverse transcription was performed at 30°C for 10 min, 42°C for 30 min, and 99°C for 5 min. The following primers were used: CCAAT/enhancer binding protein-alpha (C/EBP*α*), forward 5-CGCAAGAGCCGAGATAAAGC-3, and reverse 5-CACGGCTCAGCTGTTCCA-3; peroxisome proliferator-activated receptor-gamma (PPAR*γ*), forward 5-CGCTGATGCACTGCCTATGA-3, and reverse 5-AGAGGTCCACAGAGCTGATTCC-3. The C/EBP*α* and PPAR*γ* amplifications were performed by denaturing at 94°C for 1 min, annealing at 54°C for 1 min, extending at 72°C for 30 sec for 30 cycles, administering a final extension of ethidium bromide, and then visualizing under a UV light.

### 2.10. Statistical Analysis

The results of the tests are presented as the mean ± S.D. values for each test group using the Statistical Package for the Social Sciences (SPSS). The statistical significance between test groups was analyzed using a one-way ANOVA method. Differences were analyzed using Duncan's Multiple Range Test at the *P* < 0.05 level; their significances were also analyzed.

## 3. Results

### 3.1. Analysis of the Wild *Codonopsis lanceolata* Extract

**Figure 1:**
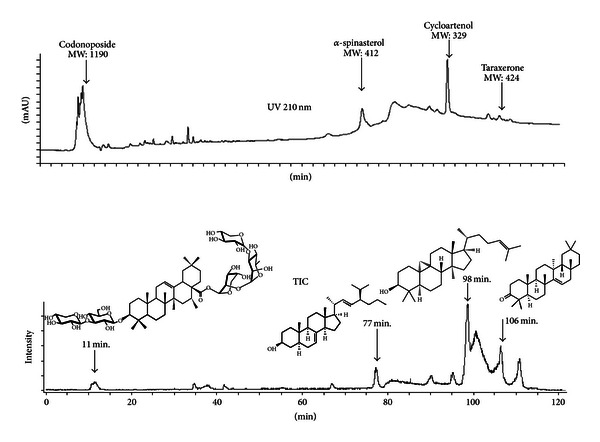
HPLC chromatogram and total ion current chromatogram of the crude extract of wCL by LC-UV-ESI-MS.

Figure 1 shows the HPLC chromatogram and the total ion current chromatogram of the total extract of wCL from the HPLC-UV/ESI-MS analysis. The protonated molecular ion of wCL (*m*/*z* 1191 ([M + H]+)) was detected at 11 min as a codonoposide, a compound known to be one of the active components of CL [24]. In addition, spinasterol, cycloartenol, and taraxerone were identified at 77 min, 98 min, and 106 min, respectively [25]. These compounds have already been reported as chemical constituents of this plant. The extracts used in this study were standardized to a codonoposide (yield: 0.3%). 

### 3.2. Body Weight and Weight Gain

Table 3 presents the influences of wCL and cCL on weight, the ingested diet amount, and the food efficiency of the rats fed with the diet. There were no particular differences between the test groups fed with the normal diet (ND) and with the normal diet supplemented with CL (ND + wCL or cCL 900 mg/kg).

However, the rats in the test group fed with the high-calorie/high-fat-diet (HFD) showed a significant increase in their weight compared to the test group fed with the normal diet (ND) (*P* < 0.01). In addition, the amount of daily ingested calories in the control group fed with the high-calorie/high-fat-diet (HFD) represented a significantly higher level compared to that of the normal diet (ND).

Interestingly, the amount of ingested diet in the groups fed with the high-fat-diet supplemented with wCL (HFD + wCL 900 mg/kg) showed a slight decrease over the high-calorie/high-fat-diet (HFD) (*P* < 0.01). There was also a significant decrease (*P* < 0.01) in weight at the high concentration (900 mg/kg) of wCL extracts, while the low (100 mg/kg) and medium (300 mg/kg) concentrations of wCL extracts and the high concentration of cCL extracts showed no significant changes in weight compared to the high-calorie/high-fat-diet (HFD) group. For the HFD + cCL groups, there were some changes in the final weight at the low and medium concentration groups, but there were increases in the food efficiency and no decreases in the ingested amount. Therefore, it can be assumed that the results are related to dehydration due to the increase in fat and the decrease in muscle rather than the decrease in body weight.

### 3.3. Influences on the Food Efficiency

To investigate the effect of test samples (sibutramine as positive control and CL) on obesity, changes in body weights and ingested food amounts were observed, and the food efficiencies were calculated from the equation outlined below (Table 3). The body weight was measured every day, and the ingested food was calculated by measuring the amount of remaining food, where food efficiency ratio (FER) = increased body weight (g)/food intake (g). 

According to this food efficiency ratio equation, a change in body weight is the most important factor affecting the food efficiency ratio, as there is no large change in the amount of ingested food. Thus, it is possible to apply the food efficiency ratio as a scale of obesity and to consider that a small value for the food efficiency ratio is also an effective parameter to predict the avoidance of obesity. When verification of diet-induced obesity using this food efficiency ratio was applied in this study, it was possible to observe that upon administration of CL (i.e., wCL or cCL) for six weeks, obesity was reduced in a concentration-dependent manner to the levels recorded in the normal diet (ND) group. The high-capacity wCL 900 mg/kg group showed the results from the ND group and the HFD group significantly lower FER. This, as showing the proper diet inhibition from all of the normal diet or the high calories diet, is regarded to be a necessary result in diet control for keeping the standard weight. In addition, the groups treated with the highest concentrations (900 mg/kg) of wCL and cCL demonstrated a decrease in the level of obesity. It can be suggested that the high concentration of CL is effective in reducing obesity. 

### 3.4. Plasma Lipid Levels and Fat Pad

**Figure 2:**
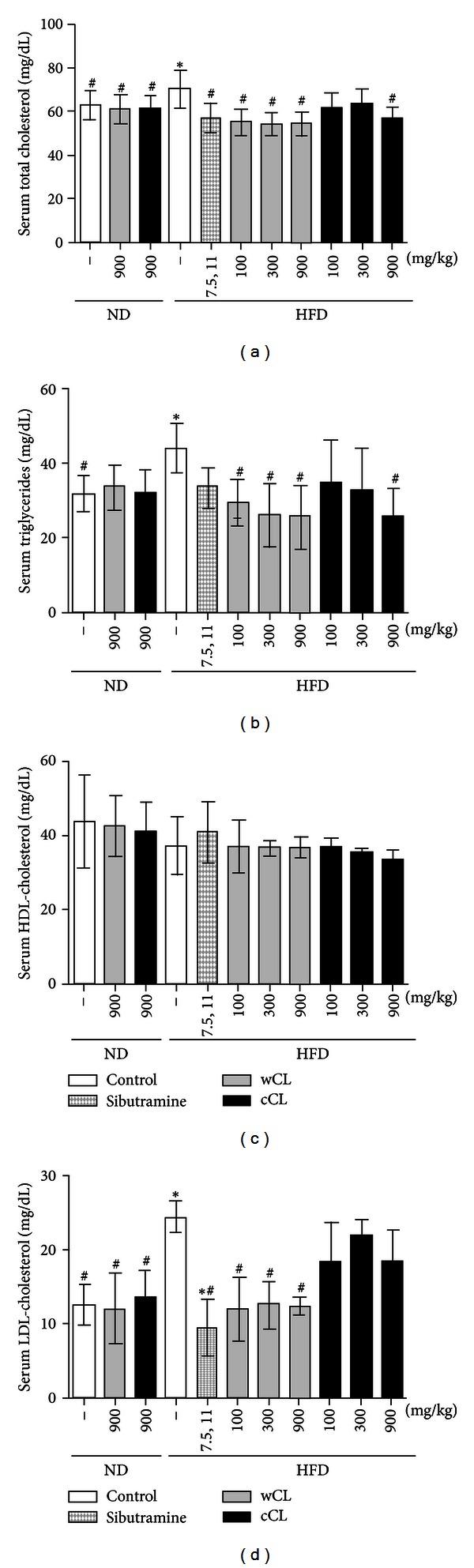
Effects of the wild and cultivated *Codonopsis lanceolata* extracts on (a) serum total cholesterol, (b) serum triglycerides, (c) serum high density lipoprotein (HDL)-cholesterol, (d) serum low density lipoprotein-(LDL-) cholesterol levels (mg/dL) in rats. Values are means ± S.D. (*n* = 10), *P < 0.05, **P < 0.01 versus ND group, ^#^ *P* < 0.05, ^##^ *P* < 0.01 versus HFD group.

The obese rats with no CL treatment showed an increase in the weight of the total fat in the abdominal cavity and in the weight of the retro peritoneum and around the epididymis in the abdominal cavity. However, the obese rats treated with the CL (i.e., wCL or cCL) showed a significant latency in the increase in weight of these fat tissues. In particular, the group treated with wCL (HFD + wCL) had a more efficient control of body weight increase in a concentration-dependent manner compared to that of the group treated with cCL (HFD + cCL). The group treated with high concentrations (900 mg/kg) of wCL showed the most significant effects on the weight of fat tissues, with the retroperitoneal fat tissues decreasing approximately 75% and the weight of epididymal fat tissues decreasing approximately 83%, compared to the high-calorie/high-fat-diet (HFD) control group. The group treated with high concentrations (900 mg/kg) of wCL also demonstrated a decrease of approximately 77% in abdominal fat levels and an increase of approximately 145% in brown adipose tissue levels, which facilitates thermal production (Table 4). Among the test groups treated with CL (HFD + CL), the group treated with the medium concentration (600 mg/kg) showed the smallest effects. To investigate the effects of wCL on the rats fed with the high-calorie/high-fat-diet (HFD) in more detail, the effects on serum triglycerides (TG), total cholesterol (TC) and high density lipoprotein-(HDL-) cholesterol levels in the rats were measured (Figure 2). Additionally, the serum fat concentrations of the rats with diet-induced obesity were measured. From the results of the measurements, the HFD control group (i.e., not treated with CL) showed high values in serum TG and TC compared to those of the normal diet (ND) group. It was evident that the HFD resulted in obesity and high cholesterol levels in rats subjected to this diet. The administration of CL significantly suppressed the increase of these levels. In the case of the TG levels, these effects were observed to be concentration dependent. 

### 3.5. T3-L1 Cells Differentiation

Adipocytes adjust their own growth and development, not only for lipid metabolism but also for the performance of functions to maintain a consistent supply of energy in the body [26].

**Figure 3:**
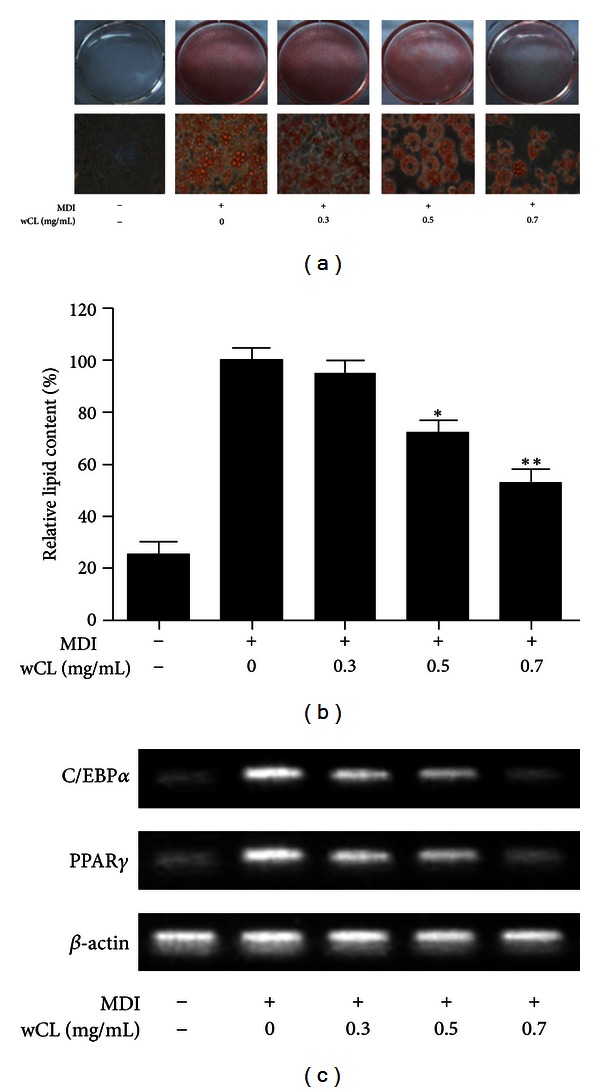
The effect of wCL on 3T3-L1 adipocyte differentiation. (a) 3T3-L1 cells were differentiated with MDI in the absence or presence of wCL (0, 0.3, 0.5, 0.7 mg/mL) for eight days, followed by measurement of lipid contents by Oil Red O staining. (b) Stained oil droplets were dissolved with isopropanol and quantified by spectrophotometric analysis at 510 nm. The results were represented as relative lipid contents. *P < 0.05, **P < 0.01 by SPSS compared to MDI-treated cells. (c) Expression of C/EBP*α* and PPAR*γ* mRNA from 3T3-L1 cells as described in (a) was measured by RT-PCR.

In this study, we observed the effects of wCL on adipocyte differentiation by a cell-based in vitro experiment with Oil Red O dye. The results shown in Figure 3 confirmed that 3T3-L1 fat generation is suppressed with treatment of wCL in a concentration-dependent manner. A similar concentration-dependent effect of wCL on the triglyceride levels in the blood samples confirmed the strong relationship between adipocyte differentiation and triglyceride levels in the blood (Figure 2). Because the level of triglycerides in serum increases with increases in the obesity index and plays an important role in lipid metabolism, it may be considered to be a major determinating parameter for obesity. To investigate the possible molecular mechanism of wCL on the suppression of obesity, the levels of CCAAT/enhancer binding protein *α* (CEBP*α*) mRNA and peroxisome proliferator-activated receptor *γ* (PPAR*γ*) mRNA were monitored in 3T3-L1 cells treated with increasing concentrations of wCL. It was observed that the expression of CEBP*α* mRNA, one of the most important transcription factors in cell differentiation, decreased with increasing concentrations of wCL and the expression of PPAR*γ* mRNA, which plays an important role in promoting storage of fatty acids in adipose tissue, also decreased with wCL treatments in a concentration-dependent manner.

## 4. Discussion

It is widely accepted that the levels of saturated fat and cholesterol in the diet increase the level of serum cholesterol, while a diet with a low fat content decreases the level of serum cholesterol. There have been tremendous efforts to find efficient food sources from traditional herbal medicines that can be used to optimize the level of serum cholesterol, thereby controlling obesity. Ginseng is one of the most promising candidates for a functional food of this type. It has been reported that the saponin in ginseng increased the absorption of LDL in the liver and facilitated the removal rate of abnormally increased VLDL by the intake of high levels of cholesterol from the diet [27]. It has also been reported that the decrease in the level of blood cholesterol by the administration of saponin derived from ginseng was mainly dependent on the decrease in the concentrations of VLDL and LDL in serum. In this present study, it was also assumed that the significant decreases in the total blood cholesterol of all groups fed with HFD and treated with wCL were due to the action of the saponins in the wCL, as saponins have been reported as active components of CL [28]. Although the uniform effects on the levels of serum cholesterol and lipids were obtained in the group treated with the highest concentration level of wCL (HFD + wCL 900 mg/kg), there were small improvements in the fat and blood lipid levels in the group treated with cCL (HFD + cCL). The reason that wCL water extracts showed more effective results than cCL extracts is likely due to differences in the content and types of active ingredients between wCL and cCL products.

Mitochondrial *β*-oxidation of long-chain fatty acids is the major source of energy. Prior to undergoing *β*-oxidation in mitochondrial matrix, the long-chain fatty acyl-CoA must be transferred from cytosol into matrix. The lipid metabolism related many enzymes in this process. The present study has investigated the effects of wCL on lipid and cholesterol metabolisms in normolipidemic and hyperlipidemic rat. We were able to get the results, wCL inhibiting activity of fat oxidation.

The cholesterol lowering of property wCL could be due to increased excretion of cholesterol and bile acids. These observed effects can be attributed to the presence of phytosterol in wCL as phytosterols possesse greater affinity for micelles than cholesterol and reduce incorporation of cholesterol in micelles [29]. We can see from the configuration that the phytochemical components of this wCL are composed of the enzyme controlling largely physiological vitality of adipose metabolism and joining matters.

The effect of CL extract on obesity control does not seem to be related to a direct inhibition of the neurotransmitter cascade, the main mechanism of sibutramine and a serotonin-norepinephrine reuptake inhibitor. The treatment with wCL resulted in the control of serum lipid levels, a decrease in the content of the total body fat, and a significant increase in the level of brown adipose tissue. However, sibutramine-treated models (HFD + sibutramine) have shown that the obesity control effect does not accompany an increase in the brown adipose tissue that is known to have high capability in fat burning and fatty acid oxidation. Direct action of CL on the lipid levels in serum, rather than a central control of appetite, could exclude various side effects in the central nervous system (CNS) found in antiobesity agents such as sibutramine. 

Growth of adipose tissues is broadly divided into two stages: the increase in fat cell size and the differentiation of the adipocyte from the preadipocyte [30]. During differentiation from precursor adipocytes into fully mature adipocytes, either ecologically or biochemically, differentiating factors are activated, which are important for regulating the adipocyte genes [30, 31]. 

Various transcription factors like PPAR*γ* and C/EBP family are involved during adipocyte differentiation. Among them, the expression of PPAR*γ* and C/EBP*α* in 3T3-L1, which is a wellknown and frequently used preadipocyte cell line for in vitro adipocyte differentiation [32], is upregulated [33]. PPAR*γ* and C/EBP*α*, the key adipogenic and lipogenic transcriptional regulators, are crucial to the regulation of obesity and adipocyte differentiation [34]. Recently, studies have shown that PPAR*γ* is necessary and sufficient to promote adipogenesis and that C/EBP*α* is influential in maintaining the expression of PPAR*γ* [35–37]. The results of our study demonstrate that the treatment with wCL extract suppresses the accumulation of triglycerides within the cells and the differentiation of 3T3-L1 preadipocytes into adipocytes. Total CL group significantly reduced fat accumulation by inhibiting adipogenic signal transcriptional factors, such as PPAR*γ* and C/EBP*α* mRNA, which functions via AMPK (5′ AMP-activated protein kinase or 5′adenosine monophosphate-activated protein kinase) signaling, in vitro. In other words, the concentration-dependent suppression of C/EBP*α* and PPAR*γ* mRNA expression in CL-treated cells represented one of the possible mechanisms of the suppression of 3T3-L1 cells differentiation.

AMPK is an enzyme that plays a role in cellular energy homeostasis. The net effect of AMPK activation is stimulation of hepatic fatty acid oxidation and ketogenesis, and inhibition of cholesterol synthesis, lipogenesis, and triglyceride synthesis [38]. AMPK and PPAR*γ* appear to be involved in adipocyte differentiation and maturation and thus can be potential drug targets for the treatment of obesity. wCL significantly inhibited the expression levels of C/EBP*α* and PPAR*γ*, two master regulators of adipogenesis (Figure 3), indicating that wCL might inhibit 3T3-L1 differentiation via suppressing the expression of adipogenesis-related transcription factors and markers. Meanwhile, PPAR*γ* transcriptional activity was reduced supporting the wCL downregulated PPAR*γ* expression as well as its transcriptional activity. AMPK phosphorylates the transcriptional coactivator p300 and induces its interaction with PPAR*γ* [33]. It was supposed that wCL might suppress PPAR*γ* transcriptional activity via activating AMPK and phosphorylating the transcriptional coactivators and hence leading to the inhibition of their abilities to interact with PPAR*γ*.

In conclusion, we can observe that wCL improved the restraint of excessive adipose formation and accumulation by these matters. The potential of CL as a promising source for the development of a functional food, which can be efficiently used to control obesity, should be appreciated and evaluated with more detailed molecular studies. 

## Figures and Tables

**Table 1:** Composition of the normal diet and the high-calorie/high-fat diet (g/100 g).

Groups ingredient	Normal diet	High-calorie/high-fat diet*
Casein	20.0	29.0
Corn starch	60.0	10.0
Sucrose	—	10.0
Corn oil	9.0	5.0
Cellulose	5.0	5.0
Lard	—	35.0
AIN-76 mineral mix	3.5	3.5
AIN-76 vitamin mix	1.0	1.0
DL-Methionine	0.3	0.3
Choline bitartrate	0.2	0.2

kcal/100 g diet	390.2	458.0
Calorie from fat (%)	11.5	35.0

*High-calorie/high-fat diet was modified from the AIN-76 dietary composition [21].

**Table 2:** Classification of experimental groups.

Group	Treatment
ND	Normal diet
ND + wCL 900 mg/kg	ND + wild *Codonopsis lanceolata* 900 mg/kg/day
ND + cCL 900 mg/kg	ND + cultivated *Codonopsis lanceolata* 900 mg/kg/day
HFD	High-calorie/high-fat diet
HFD + sibutramine	HFD + sibutramine 7.5, 11 mg/kg/day
HFD + wCL 100 mg/kg	HFD + wild *Codonopsis lanceolata *100 mg/kg/day
HFD + wCL 300 mg/kg	HFD + wild *Codonopsis lanceolata *300 mg/kg/day
HFD + wCL 900 mg/kg	HFD + wild *Codonopsis lanceolata *900 mg/kg/day
HFD + cCL 100 mg/kg	HFD + cultivated *Codonopsis lanceolata *100 mg/kg/day
HFD + cCL 300 mg/kg	HFD + cultivated *Codonopsis lanceolata *300 mg/kg/day
HFD + cCL 900 mg/kg	HFD + cultivated *Codonopsis lanceolata* 900 mg/kg/day

**Table 3:** Effects of water extracts of wild and cultivated *Codonopsis lanceolata *on body weight, food efficiency ratio, and food intake in SD rats fed with normal or high-fat diets.

Group	Body weight (g)		Food efficiency ratio^1^	Food intake (g/day)
	Initial	Final		
ND	213.7 ± 10.11	481.60 ± 35.68^##^	0.21 ± 0.03^#^	57.24 ± 8.17^#^
ND + wCL 900 mg/kg	213.6 ± 8.97	473.40 ± 26.38^##^	0.17 ± 0.05^∗##^	55.14 ± 10.06^#^
ND + cCL 900 mg/kg	213.6 ± 8.11	484.20 ± 26.15^##^	0.22 ± 0.04	57.47 ± 12.33^#^
HFD	213.6 ± 7.89	546.80 ± 36.84**	0.24 ± 0.05*	71.46 ± 6.80*
HFD + Sibutramine	213.6 ± 7.87	474.00 ± 30.20^##^	0.14 ± 0.03^∗∗##^	42.72 ± 6.56^∗##^
HFD + wCL 100 mg/kg	213.6 ± 7.66	516.22 ± 32.73*	0.21 ± 0.05^#^	52.60 ± 5.42^#^
HFD + wCL 300 mg/kg	213.6 ± 7.50	524.22 ± 58.10*	0.22 ± 0.07	49.92 ± 11.57^##^
HFD + wCL 900 mg/kg	213.6 ± 7.70	499.75 ± 30.32^#^	0.17 ± 0.08^∗##^	46.35 ± 10.68^∗##^
HFD + cCL 100 mg/kg	213.6 ± 7.55	504.20 ± 31.20^#^	0.26 ± 0.06*	48.85 ± 4.38^##^
HFD + cCL 300 mg/kg	213.6 ± 7.55	509.33 ± 36.86^#^	0.22 ± 0.04	47.37 ± 6.50^##^
HFD + cCL 900 mg/kg	213.6 ± 7.05	520.75 ± 22.52*	0.17 ± 0.08^∗##^	50.28 ± 9.09^##^

^1^Food efficiency ratio (FER) = increased body weight (g)/Food intake (g).

Values are the means ± S.D. (*n* = 10), *P < 0.05, **P < 0.01 versus ND group, ^#^ *P* < 0.05, ^##^ *P* < 0.01 versus HFD group.

**Table 4:** Effects of water extracts of wild and cultivated *Codonopsis lanceolata *on retroperitoneal, epididymal, brown fat, and total abdominal fat in SD rats fed with normal or high-fat diets.

Group	Retroperitoneal (g)	Epididymal (g)	Brown fat (g)	Total abdominal (g)
ND	6.21 ± 3.30^##^	9.40 ± 4.00^##^	0.43 ± 0.08	13.80 ± 4.05^##^
ND + wCL 900 mg/kg	5.89 ± 1.69^##^	9.76 ± 2.63^##^	0.54 ± 0.16	14.50 ± 2.92^##^
ND + cCL 900 mg/kg	6.24 ± 1.20^##^	9.95 ± 3.36^##^	0.52 ± 0.11	14.58 ± 3.09^##^
HFD	11.01 ± 1.79**	15.19 ± 2.57**	0.44 ± 0.10	25.78 ± 3.67**
HFD + Sibutramine	6.81 ± 1.54^##^	11.16 ± 3.89^#^	0.33 ± 0.07	18.60 ± 2.86^∗#^
HFD + wCL 100 mg/kg	8.88 ± 1.20^∗#^	15.77 ± 4.34**	0.40 ± 0.07	23.26 ± 3.12**
HFD + wCL 300 mg/kg	8.69 ± 0.83^∗#^	13.42 ± 1.36*	0.53 ± 0.12	21.94 ± 4.84**
HFD + wCL 900 mg/kg	8.25 ± 1.69^∗#^	12.67 ± 3.79^∗#^	0.64 ± 0.10^∗∗##^	19.91 ± 4.46^∗#^
HFD + cCL 100 mg/kg	9.61 ± 2.28*	13.12 ± 2.01*	0.54 ± 0.07	20.73 ± 5.21^∗#^
HFD + cCL 300 mg/kg	10.60 ± 2.23*	14.58 ± 3.10**	0.67 ± 0.11^∗∗##^	24.23 ± 5.71**
HFD + cCL 900 mg/kg	9.99 ± 3.10*	14.10 ± 3.81**	0.56 ± 0.20**	22.82 ± 3.23**

Values are means ± S.D. (*n* = 10), *P < 0.05, **P < 0.01 versus ND group, ^#^ *P* < 0.05, ^##^ *P* < 0.01 versus HFD group.
